# Public administration and health system strengthening in low- and middle-income countries: exploring strategies to improve healthcare delivery and health governance

**DOI:** 10.3389/frhs.2026.1798165

**Published:** 2026-05-14

**Authors:** James Ndlovu, Zamokuhle Mbandlwa

**Affiliations:** Faculty of Management Sciences, Department of Public Management and Economics, Durban University of Technology, Durban, South Africa

**Keywords:** health governance, health system strengthening, institutional capacity building, low-and middle-income countries (LMICs), public administration

## Abstract

**Introduction:**

Health system strengthening in low- and middle-income countries (LMICs) remains a persistent challenge in LMICs, where reforms often fail to translate into sustained improvements in access, quality, and equity. While existing scholarship has largely focused on technical and financing interventions, less attention has been given to the institutional and administrative conditions that shape reform outcomes. This study addresses this gap by examining how public administration influences health system performance. Guided by Institutional Theory, the study argues that HSS is fundamentally an institutional process, mediated by the coherence and effectiveness of administrative systems.

**Methods:**

A qualitative comparative case study design was adopted, focusing on three purposively selected cases: Germany (as a governance comparator), South Africa, and India. Data were collected through documentary analysis of peer-reviewed literature, policy documents, and reports from international organisations. The analysis employed a theoretically informed thematic approach, combining inductive coding with deductive categories derived from Institutional Theory. Comparative analysis was conducted across key dimensions, including governance arrangements, decentralisation, leadership capacity, and digital health integration.

**Results:**

The findings reveal that institutional capacity and governance coherence are decisive in shaping health system outcomes. Germany demonstrates how aligned governance structures and regulatory mechanisms enable consistent policy implementation. In contrast, South Africa and India exhibit institutional fragmentation, uneven decentralisation outcomes, and variable leadership capacity, which constrain reform effectiveness. Decentralisation produces divergent outcomes depending on local institutional strength, while digital health integration remains uneven due to infrastructural and administrative limitations. Across cases, the interaction between governance, leadership, and institutional capacity emerges as a critical determinant of reform success.

**Discussion:**

The study demonstrates that public administration is not a peripheral factor but a central explanatory variable in HSS. The findings support and refine prior work on governance and health systems by showing that institutional alignment, rather than policy design alone, determines implementation outcomes. Theoretically, the study advances Institutional Theory by illustrating how formal and informal administrative arrangements shape health system performance. Practically, it highlights the need for context-sensitive reforms that prioritise institutional strengthening, leadership development, and coordinated governance. These insights contribute to both public administration and health policy debates, emphasising the necessity of integrating institutional analysis into future health system reforms in LMICs.

**Conclusion:**

This study contributes to a deeper understanding of how public administration practices can foster sustainable health system strengthening in LMICs.

## 1 Introduction

Health system strengthening (HSS) has become a central concern in public policy and development scholarship because many low- and middle-income countries (LMICs) continue to face persistent deficits in service access, quality, equity, and system resilience despite repeated rounds of reform (1, 2). In this article, health system strengthening is understood as the deliberate process of improving the core functions, institutions, and relationships that shape health service delivery, financing, governance, and accountability so that health systems can produce better and more equitable outcomes over time (1, 3). On the other hand, public administration is conceptualised here not simply as the machinery of government, but as the set of formal rules, organisational arrangements, leadership practices, regulatory mechanisms, and implementation capacities through which public authority is exercised in the health sector. This includes how decisions are coordinated, how resources are allocated, how actors are held accountable, and how policy is translated into routine service delivery (4, 5). Framing the problem in this way is important because weak health outcomes in LMICs often reflect not only shortages of money, infrastructure, or personnel, but also deficits in governance, administrative capability, and institutional coherence that undermine reform implementation (6, 7).

Theoretically, this paper is underpinned by Institutional Theory, which provides the central analytical lens for examining how health systems are shaped by formal structures, normative expectations, and embedded administrative practices. Institutional theory is relevant to HSS because it draws attention to the fact that policy outcomes are not determined by technical design alone; they are also conditioned by the quality of institutions that organise authority, regulate behaviour, and structure relationships among state agencies, professionals, and communities (3, 4). In LMIC contexts, this perspective is especially useful because governance failures are frequently rooted in weak institutional arrangements, fragmented authority, poor accountability systems, and limited capacity for implementation rather than in the absence of policy intent (8, 9). Guided by this framework, the paper argues that health system strengthening in LMICs is fundamentally an institutional and administrative challenge as much as it is a technical or financial one. More specifically, the paper contends that stronger health outcomes are more likely where public administration is able to provide coherent governance, capable leadership, effective regulation, intersectoral coordination, and context-sensitive implementation capacity. The paper's scientific contribution lies in bringing these dimensions together within a theoretically informed comparative analysis, thereby showing how institutional arrangements mediate the success or failure of HSS efforts across contrasting settings.

The motivation for this study arises from an important gap in the literature. Existing scholarship on HSS in LMICs is extensive, but much of it has focused either on technical interventions, disease-specific programmes, financing reforms, or primary healthcare models, often without sufficient attention to the administrative and institutional conditions that make such reforms work in practice (10, 11). Similarly, research on governance has demonstrated its importance for health outcomes, yet the evidence base remains uneven, with persistent gaps regarding how public administration functions operate across different LMIC contexts and how these functions can be comparatively interpreted through a coherent theoretical lens (4, 12). Studies have also noted the need to ask better evaluative questions about HSS interventions, especially questions that move beyond programme effectiveness toward understanding the systems, institutions, and policy environments in which interventions are embedded (6). This paper responds to this gap by examining public administration not as a background variable, but as a primary explanatory factor in health system performance. In doing so, it contributes to the literature by clarifying the institutional mechanisms through which governance, decentralisation, leadership, and administrative capacity shape system strengthening efforts.

The research is also relevant because contemporary health systems in LMICs are under pressure from multiple and overlapping demands: the unfinished agenda of primary healthcare, rising non-communicable disease burdens, fiscal constraints, technological transitions, and the need to improve resilience in the face of shocks and crises (13–15). These pressures make it increasingly important to understand why some public systems are able to coordinate reform and sustain implementation while others remain fragmented or ineffective. To comprehensively address this question, the paper adopts a qualitative comparative case study design, because such an approach is well suited to examining how governance arrangements operate in context and how administrative processes interact with policy implementation in different institutional environments (6, 8). Rather than seeking statistical generalisation, the study aims for analytical depth and structured comparison. The selected cases were chosen purposively to capture variation in governance traditions, institutional capacity, and reform experience across settings that illuminate the article's core argument. In line with this logic, the article focuses on one European case used as a governance comparator, one African LMIC case, and one Asian LMIC case. Europe is included not as a universal model, but as a useful benchmark for examining how governance design and administrative coordination influence primary healthcare delivery (16). The African case speaks directly to the persistent governance and implementation constraints that characterise many LMIC health systems, including dependence on external financing and capacity limitations (17). The Asian case, meanwhile, provides insight into how administrative reform strategies are being pursued under conditions of fiscal pressure and service delivery inefficiency (18, 19). This case selection therefore supports meaningful comparison across different institutional environments while keeping the analysis sufficiently bounded and policy relevant.

Against this background, the article asks how public administration shapes health system strengthening in LMICs and what institutional lessons can be drawn from comparative analysis for future reform. Its contribution is threefold: firstly, it advances a clearer conceptualisation of the relationship between public administration and HSS. Secondly, it applies Institutional Theory to demonstrate why governance structures and administrative capacities are central to reform outcomes. Thirdly, it generates policy-relevant insights on leadership, regulation, decentralisation, and implementation that can inform ongoing efforts to build stronger and more equitable health systems in LMICs (20, 21). The article, therefore, moves beyond descriptive discussion of health reforms to offer a more explicit explanation of why institutional and administrative conditions matter for health system transformation. The rest of the paper is organised as follows: Section 2 outlines the theoretical framework underpinning the study. Section 3 comprises the literature review. Section 4 presents the research methodology, outlining the process and steps undertaken to collect and analyse the data. Sections 5, 6 are dedicated to the presentation of the results as well as discussion of the findings. Lastly, Section 7 provides the conclusion of the study, summarising the key insights and implications drawn from the research.

## 2 Theoretical framework: institutional theory

In exploring public administration's role in HSS, it is essential to draw on appropriate theoretical frameworks to guide the analysis. In this paper, Institutional Theory provides a comprehensive understanding of how governance structures, leadership and accountability mechanisms shape health system performance in LMICs. Institutional Theory focuses on the structures, processes and practices that shape organizations in a society. It posits that organizational behaviour is influenced not only by economic or technical factors but also by social and political pressures that shape institutions (4). In the context of health systems, Institutional Theory helps explain how health systems are shaped by national governance structures, cultural norms, historical factors and the political environment.

This theory is particularly relevant for LMICs, where weak institutional frameworks often undermine health system effectiveness. According to Adam and de Savigny (3), institutions in LMICs are often underdeveloped, which leads to inefficient policy implementation and weak accountability mechanisms that ultimately compromise healthcare delivery. The theory highlights how health systems can be strengthened through the development of more robust institutions, including the creation of professional health leadership, effective governance frameworks and policies that support sustainable health service delivery (21).

For example, countries such as Rwanda have demonstrated that strong institutional frameworks, supported by effective leadership and clear accountability mechanisms can significantly improve health outcomes despite resource constraints (22). Institutional Theory suggests that for LMICs to enhance their health systems, they must prioritize institutional development, focusing on building regulatory frameworks and leadership capacity that support health system resilience.

## 3 Literature review

The literature on HSS in LMICs has evolved significantly, with a growing recognition that the core challenges facing these systems are not merely financial or infrastructural, but are deeply rooted in governance, institutional capacity, and leadership. These themes are essential to understanding why some LMICs struggle to implement reforms despite substantial policy efforts. In this review, key concepts such as governance, institutional capacity, decentralisation, leadership, and digitalisation are critically examined and integrated into a conceptual framework that supports the subsequent empirical analysis. By aligning these concepts within a unified theoretical structure, this section aims to develop a coherent narrative that underscores their interdependence in shaping health system performance.

### 3.1 Governance and institutional capacity in health systems

Governance in health systems refers to the processes by which authority is exercised, and decisions are made regarding policy, regulation, and the allocation of resources within health services (4). In the context of LMICs, effective governance is often characterised by weak institutional frameworks that fail to provide the necessary coordination, accountability, and regulation to ensure equitable and efficient healthcare delivery (5). Institutional capacity thus becomes a pivotal concept in understanding governance in LMIC health systems. It is defined as the ability of health institutions to perform core functions such as policy implementation, resource management, and service delivery (6).

The quality of governance mechanisms directly impacts health outcomes in LMICs. Strong governance structures, supported by effective institutions, tend to foster accountability, transparency, and equity, leading to better service delivery (23). However, many LMICs experience weak governance due to fragmented authority, political instability, and poor regulatory mechanisms that hinder system performance (8). This is evident in South Africa, where despite ambitious reforms like the National Health Insurance (NHI) system, poor coordination between national and local authorities has led to service delivery disparities (22). Similarly, in Uganda, decentralisation efforts have resulted in resource inequities due to weak local governance and insufficient capacity at the district level (21).

This highlights the central role of institutional capacity building in HSS. Institutional capacity in health systems is not just about the availability of resources, but also about developing the administrative systems necessary to manage those resources effectively. As Bennett et al. (20) argue, building institutional capacity requires not only training health managers and leaders but also strengthening governance frameworks, enhancing health information systems, and improving regulatory mechanisms to ensure sustained improvements in healthcare delivery.

### 3.2 Decentralisation and its influence on health systems

Decentralisation is often considered a solution to governance inefficiencies in LMICs. By transferring decision-making powers and resources to lower levels of government, decentralisation aims to make health services more responsive and tailored to local needs (24). In practice, however, decentralisation's impact is mixed, depending on the strength of local governance systems, the adequacy of resources, and the clarity of policy coordination between central and local authorities (21).

In countries like Rwanda, decentralisation has been a success story, as strong local governance structures and coordinated health policies have led to improved service delivery and more equitable resource distribution (22). In contrast, Uganda has struggled with decentralisation, where local governments lack the technical capacity and funding to manage health services efficiently, resulting in uneven service quality across regions (21). Decentralisation in these contexts can also exacerbate service delivery fragmentation, especially when local governance structures are not sufficiently strengthened to manage newly devolved responsibilities (2).

The literature suggests that decentralisation can lead to positive outcomes when paired with institutional capacity building at the local level, ensuring that local governments have the tools, resources, and support needed to manage healthcare effectively (4). A centralised model of governance, as seen in Germany and the United Kingdom (UK), can provide a counterpoint to decentralisation by ensuring uniform policies and equitable distribution of services across regions (16). However, decentralisation remains a valuable tool for enhancing local responsiveness, provided that governance structures and accountability mechanisms are sufficiently strong to avoid inequalities (20).

### 3.3 Leadership and governance capacity

Leadership in health systems refers to the ability of health administrators and policymakers to set clear directions, make strategic decisions, and lead reforms. In many LMICs, leadership is an essential factor in overcoming governance failures and implementing health reforms effectively. Strategic leadership is particularly important in environments marked by political instability, resource constraints, and institutional fragmentation, as leaders must navigate these challenges while ensuring that health policies are responsive to the needs of the population (21, 22).

Effective leadership in public administration is closely tied to governance capacity, which enables health systems to adapt to changing health needs and demands. Leadership capacity building involves not only improving technical expertise but also fostering collaboration, accountability, and transparency within the health system (5). In South Africa, for example, despite significant investment in health infrastructure, leadership failures in coordinating national and provincial efforts have undermined the effectiveness of reforms such as the NHI (22). Similarly, countries like Malawi and Syria demonstrate how weak leadership and poor governance can lead to poor health outcomes and slow reform implementation (15, 17).

Thus, strong leadership is essential for the successful implementation of health reforms, as it enables leaders to build trust, manage intersectoral coordination, and align health system goals with broader development priorities (22). Leadership development, therefore, should be a central component of institutional capacity building in LMICs, aimed at improving the quality of governance and the delivery of health services.

### 3.4 Digital health and technology integration

In the era of digital health, the integration of digital technologies such as telemedicine, electronic health records (EHRs), and mobile health (mHealth) applications is increasingly seen as an essential strategy for improving health system access and efficiency (25, 26). Digital health solutions have the potential to overcome some of the major health system constraints faced by LMICs, including geographic barriers, resource shortages, and service fragmentation (27). Countries like Estonia and the UK have demonstrated that digital health tools can significantly improve healthcare coordination, reduce administrative costs, and enhance accessibility, particularly in rural areas (26).

However, in LMICs, the digital divide remains a significant challenge. Barriers to digital health integration include inadequate infrastructure, low digital literacy, and insufficient funding for technology adoption (14). Despite these challenges, Kenya and India have made strides in integrating mobile health technologies to improve maternal health and disease monitoring (14, 28). Telemedicine in particular has proven effective in remote consultations and disease management, offering significant opportunities for improving access to care in underserved populations (25).

For digital health solutions to be successfully integrated into health systems in LMICs, they must be designed with consideration of the local context, addressing infrastructure gaps and digital illiteracy while aligning with broader health system goals (14). The experience of countries like Saudi Arabia, which has invested heavily in telemedicine and EHR systems, offers a model for other LMICs aiming to leverage digitalisation to improve health system efficiency (18).

This available literature demonstrates that health system strengthening in LMICs is inextricably linked to institutional and administrative factors, including governance, leadership, decentralisation, and digital health integration. These elements must be considered as part of a holistic governance framework that takes into account both formal structures (such as regulations, policies) and informal practices like leadership and accountability. Institutional Theory provides a coherent framework for understanding how these factors influence health system outcomes. The theory highlights the importance of institutional capacity in enabling effective governance and policy implementation (3). The literature suggests that LMICs should focus on strengthening institutional frameworks, improving leadership capacity, and integrating digital health technologies to build more resilient and effective health systems.

This review establishes a clear conceptual foundation for understanding how public administration shapes health system performance in LMICs. By synthesising the key concepts of governance, decentralisation, institutional capacity, leadership, and digital health, it provides a comprehensive framework for the empirical analysis that follows. The literature reviewed underscores the interdependence of these concepts and suggests that improvements in health systems will only occur when these elements are aligned and effectively implemented within the specific regional contexts of LMICs. This theoretical understanding will guide the comparative case study analysis and provide insights for policy formulation and health system reform.

## 4 Methodology

This study adopts a qualitative research design, using comparative case studies to examine the role of public administration in strengthening health systems in low- and middle-income countries (LMICs). A qualitative approach allows for an in-depth exploration of governance structures, leadership practices, decentralisation processes, and digital health integration within different LMIC contexts. The comparative case study method is particularly well-suited for this research because it enables the detailed analysis of health system performance across diverse settings, thus providing insights into the factors that contribute to successful health system strengthening and the institutional conditions that shape reform outcomes.

### 4.1 Case study selection

The selection of case studies is a critical aspect of the research design, as it directly impacts the insights gained from the study. The cases included in this study were selected purposively based on specific criteria aimed at capturing variation across governance traditions, institutional capacity, and health system reforms. The cases represent a diverse cross-section of LMICs, allowing for a nuanced understanding of the ways in which public administration influences health system strengthening in different regional contexts.

The criteria for selecting the case studies are as follows:

1. Geographic Diversity: The study examines cases from Europe, Africa, and Asia, providing insights into how different regions have approached health system strengthening under varied political, economic, and social conditions.
2. Variation in Governance and Institutional Capacity: The selected countries differ in terms of governance models (centralised vs. decentralised), institutional capacity (strong vs. weak health institutions), and policy reforms (successful vs. challenged reforms). This variation allows for a comprehensive understanding of the factors that influence health system performance.
3. Relevance to Health System Strengthening: The countries selected have engaged in significant health system reforms or are in the process of implementing key reforms, such as decentralisation or digital health initiatives, making them relevant for the research focus on public administration's role in HSS.

In total, three case studies were selected, each representing a distinct LMIC context:

- Germany (Europe): Chosen as a comparator for centralised governance and high institutional capacity in health system performance.
- South Africa (Africa): Selected for its ambitious reforms like the National Health Insurance (NHI) and its challenges in decentralisation and institutional coordination.
- India (Asia): Included for its ongoing digital health integration and fiscal constraints that influence healthcare delivery.

### 4.2 Data collection

The data for this study were collected through documentary analysis and the review of secondary sources. Given the focus on public administration and governance, the primary sources of data included academic literature, government reports, policy documents, and grey literature from international organisations such as the World Health Organization (WHO), the World Bank, and national health ministries.

The types of documents analysed include:

- Health policy documents: These include official government health strategies, reforms, and reports on health system performance.
- Reports from international organisations: Documents from the WHO, World Bank, and other global health bodies that provide assessments of health system performance, policy implementation, and recommendations for improvement.
- Peer-reviewed articles: Published research on governance, leadership, decentralisation, and health system strengthening in the selected countries.
- Grey literature: Non-peer-reviewed documents, including reports from NGOs and think tanks, which provide context-specific insights into health system governance.

The time period considered for document analysis spans from 2000 to the present, ensuring that both historical and recent data are included. This time frame allows for the examination of long-term trends in health system governance and reform, as well as an evaluation of the impact of more recent policy interventions.

**Inclusion criteria** for the documents were as follows:

- Documents must focus on health system governance, decentralisation, institutional capacity, or leadership in the context of LMICs.
- Documents must be relevant to the selected case study countries.
- Both peer-reviewed and grey literature were included to provide a balanced view of the data, encompassing both academic and policy perspectives.

**Exclusion criteria** included:

- Documents that were outdated or did not address the specific themes related to public administration and HSS.
- Documents that lacked empirical or theoretical grounding in the study of governance and public administration.

### 4.3 Data analysis

The data analysis for this study followed a thematic analysis approach, which is well-suited for qualitative research aimed at identifying, analysing, and reporting patterns (themes) within data (29). Thematic analysis allows for flexibility in exploring the key themes related to public administration and HSS, while also maintaining the rigor necessary to draw meaningful comparisons across the case studies.

The analysis process involved several stages:

- Familiarisation with the data: All documents were reviewed to gain an overall understanding of the governance structures, decentralisation efforts, leadership practices, and digital health strategies in each case study country.
- Initial coding: The data were coded inductively, with initial codes emerging directly from the documents. This process was guided by the conceptual framework based on Institutional Theory, which provided a lens for identifying key themes related to governance mechanisms, institutional capacity, and leadership.
- Theme identification: Through iterative coding, predefined themes such as governance mechanisms, institutional capacity, decentralisation, leadership, and digital health emerged from the data. These themes were used to structure the analysis and allowed for cross-case comparisons. The themes were also explored for any subthemes or nuanced patterns that could deepen the understanding of how public administration shapes health system performance.
- Category refinement: Once the initial themes were identified, they were refined to ensure they were meaningful and aligned with the research objectives. This involved revisiting the data and merging or splitting categories where necessary to improve clarity and coherence.
- Cross-case comparison: After the data were organised by theme, a comparative analysis was conducted across the three case study countries. This comparison focused on the governance models, institutional capacity, and leadership strategies employed by each country and how these influenced the implementation of health system reforms. The comparative logic was based on the dimensions of governance (centralised vs. decentralised), institutional strength, and the success or challenges in implementing reforms.

### 4.4 Ensuring credibility and validity

To ensure the credibility and validity of the results, several strategies were employed:

- Triangulation: Data were triangulated across multiple sources, including academic literature, government reports, and grey literature. This allowed for cross-validation of findings and ensured that the analysis was not based solely on one source type or perspective.
- Member checking: Where available, findings were cross-checked with experts in the field of health system governance and public administration. This helped refine the interpretations and ensured that the themes identified were consistent with expert views.
- Audit trail: A clear audit trail of the research process was maintained, documenting all decisions made during data collection, coding, and analysis. This enhances transparency and ensures that the methodology can be replicated or reviewed for rigor.
- Reflexivity: The research process was conducted with awareness of potential biases that might arise from the researcher's positionality. This included reflecting on how personal experiences or assumptions could influence the interpretation of the data. Regular discussions with colleagues and mentors were used to check for these biases and to improve the objectivity of the analysis.

### 4.5 Ethical considerations

#### 4.5.1 Ethical considerations

As this study relies on secondary data and document analysis, ethical considerations primarily involve ensuring that the sources used are reliable and that proper citations are provided for all data referenced. Given the focus on publicly available documents, there are no direct interactions with participants, and the study does not involve primary data collection. However, the study followed ethical guidelines related to transparency, citation, and the proper use of sources to avoid plagiarism and ensure the integrity of the research.

## 5 Results

This section presents the findings from the comparative analysis of health system strengthening in Germany, South Africa, and India. The analysis is guided by the conceptual framework based on Institutional Theory, with a focus on the role of public administration, governance mechanisms, decentralisation, leadership, and digital health integration in shaping health system outcomes. The results are presented according to the core themes identified through the thematic analysis, highlighting both similarities and differences across the three case studies.

### 5.1 Institutional capacity and governance

The comparative analysis reveals that institutional capacity is a key determinant of health system performance across all three case studies. However, the specific ways in which governance structures and institutional capacity manifest differ significantly between the regions.

Germany's health system is characterised by strong institutional frameworks and centralised governance, which ensure equitable resource distribution and uniform policy implementation. This centralisation is coupled with robust regulatory oversight at the national level, which enhances accountability and transparency in healthcare service delivery. Governance in Germany facilitates a coherent health policy that effectively aligns local health services with national objectives (16). The effectiveness of these mechanisms is evident in the system's consistent health outcomes, especially in terms of universal health coverage (UHC) and the efficient management of resources.

South Africa presents a contrasting case, where institutional capacity is weakened by fragmented governance and poor coordination between national and provincial health authorities. The introduction of National Health Insurance (NHI) has been a significant policy reform, but its implementation has been hindered by institutional fragmentation, particularly at the local level. Despite substantial investment in health infrastructure, governance challenges persist, resulting in inequities in service delivery, particularly in rural and underserved areas (22). The governance failures in South Africa underline the importance of integrating local governance structures into broader health system reforms.

India demonstrates a combination of decentralised and centralised governance elements. While decentralisation in some states has led to locally tailored health interventions, the overall lack of institutional capacity at the state and district levels has impeded the effectiveness of these reforms. Decentralisation in India has often led to regional disparities in service delivery, as local governments struggle with inadequate funding and technical capacity to manage healthcare services effectively (21). The central government has attempted to address these issues by streamlining regulatory mechanisms and focusing on strengthening local leadership and governance. While all three countries recognise the importance of governance and institutional capacity, Germany stands out for its strong centralised governance and well-established regulatory frameworks, whereas South Africa and India face challenges stemming from institutional fragmentation and inequities in governance at the local level.

### 5.2 Decentralisation and health system performance

The role of decentralisation in improving health system performance is a key theme that emerged from the comparative analysis, with notable differences in the impact of decentralised governance across the three case study countries.

In Germany, the decentralisation of health services has been integrated into a well-structured system that balances local autonomy with central oversight. While decentralisation is implemented at the regional level, the national government maintains control over the overall health policy, ensuring that health outcomes remain equitable across regions. This balance allows for responsive governance that adapts to local needs while maintaining national cohesion (16).

In contrast, South Africa's decentralisation efforts have been less effective. Despite the potential for locally adapted health interventions, decentralisation in South Africa has often resulted in disparities between provinces, with rural areas particularly disadvantaged due to weak local governance and limited resources (22). The lack of coordinated policy implementation and institutional capacity at the provincial and local levels has led to inefficiencies and unequal access to healthcare services, highlighting the need for stronger local governance structures to support decentralisation.

In India, decentralisation has had mixed results. While states with strong governance frameworks have managed to tailor health services to local needs, decentralisation has not always led to improvements in health outcomes due to the weak administrative capacity at the district level (21). Some states have implemented decentralised health policies successfully, while others continue to struggle with resource allocation and local leadership deficits.

Overall, the comparative analysis suggests that decentralisation can improve health system performance when coupled with strong local governance, institutional capacity, and intergovernmental coordination. However, in the absence of these supportive conditions, decentralisation may exacerbate existing health inequalities.

### 5.3 Leadership and policy implementation

Leadership is identified as a critical factor in driving effective health system reforms, and the comparative analysis highlights how leadership quality influences the success or failure of health policies in the three case study countries.

Strong political leadership in Germany has facilitated the effective implementation of health reforms, particularly in terms of regulatory frameworks, resource distribution, and service delivery. The alignment between policy-makers, health administrators, and service providers ensures that health policies are consistently implemented across the country, leading to high-quality care and accessibility for all citizens (16).

In South Africa, leadership challenges have been a major barrier to successful health reforms. Despite significant policy efforts, leadership fragmentation between the national and provincial levels has hindered the implementation of key reforms such as the NHI. The lack of strong political commitment and coordination has contributed to inequities in the delivery of health services, especially in marginalised areas (22).

Leadership in India has shown both strengths and weaknesses. While national leadership has driven major health reforms such as the National Health Mission, the capacity of state-level leadership to implement these policies has been inconsistent. Leadership quality varies significantly across states, with some regions showing strong administrative capacity and others struggling with political instability and poor policy implementation (21).

Strong and consistent leadership is crucial for the successful implementation of health reforms. Countries like Germany, with strong leadership at both the national and regional levels, have been more successful in translating policies into tangible outcomes compared to South Africa and India, where leadership fragmentation and inconsistent commitment to reforms have limited success.

### 5.4 Digital health integration

The integration of digital health solutions was another central theme in the comparative analysis, with a focus on the role of telemedicine, electronic health records (EHRs), and mobile health (mHealth) applications in strengthening health systems.

Germany has been a leader in integrating digital health technologies, particularly in the use of EHRs and telemedicine, which have enhanced care coordination and patient management. The widespread adoption of these technologies has resulted in efficiencies in healthcare delivery and improved access to care, especially in rural regions (26). The centralised governance structure has enabled Germany to implement nationwide digital health strategies effectively.

South Africa's adoption of digital health solutions has been slow, due to resource constraints, digital illiteracy, and infrastructural barriers. However, mobile health applications have shown potential in addressing maternal health and chronic disease management, particularly in rural areas. Despite these advances, the digital divide continues to limit the widespread adoption of digital health technologies, preventing many populations from benefiting from these innovations (14).

In India, the adoption of mHealth and telemedicine has been significant in addressing healthcare access in remote regions. However, challenges remain in terms of infrastructure gaps, digital illiteracy, and internet connectivity (28). Mobile health has shown promise in improving maternal health and disease prevention, but these benefits are not yet universally accessible across the country.

The findings underscore that digital health integration can improve health system performance when supported by adequate infrastructure, digital literacy, and government commitment to implementing technology-based solutions. The experience of Germany offers valuable lessons for South Africa and India, where the potential of digital health remains underutilised.

### 5.5 Comparative analysis and structural differences

The comparative analysis reveals several common patterns and structural differences between the reviewed case studies. Germany stands out for its centralised governance, strong institutional capacity, and successful implementation of digital health solutions. South Africa faces significant challenges in governance fragmentation, decentralisation, and leadership coordination, which hinder the successful implementation of reforms. India exhibits a mixed model of decentralised and centralised governance, with significant regional disparities in leadership quality and digital health adoption. In all cases, the findings demonstrate that institutional capacity, governance models, and leadership quality play a critical role in shaping health system outcomes. While decentralisation and digital health solutions have shown promise, their success is contingent upon strong governance frameworks, coordinated leadership, and robust institutional structures.

## 6 Discussion

This section interprets the findings from the comparative case studies of Germany, South Africa, and India, focusing on the key factors influencing health system strengthening in low- and middle-income countries (LMICs). The discussion is framed by the conceptual lens of Institutional Theory, which underscores the critical role of governance, leadership, institutional capacity, decentralisation, and digital health integration in shaping the success of health reforms. While the findings confirm several established trends in the literature, they also contribute new insights into how these concepts interact and influence health system performance in LMICs.

### 6.1 Institutional capacity and governance mechanisms

The findings reaffirm that institutional capacity is a fundamental determinant of health system performance, which aligns with previous studies that emphasise the need for strong governance frameworks to support health reforms (3, 5). In Germany, the centralised governance model, supported by well-established regulatory systems, allowed for equitable resource distribution and the effective implementation of health policies (16). This experience contrasts sharply with South Africa and India, where institutional fragmentation and weak coordination have hindered the effectiveness of reforms. South Africa's NHI, despite its promising goals, has faced substantial institutional challenges at both national and provincial levels, which have led to service delivery inequities (22).

The findings also support the argument that health system strengthening in LMICs requires not only financial investment but also institutional development (7). This is particularly evident in India, where decentralisation efforts have been undermined by weak local governance and inadequate institutional capacity at the district level. The mixed governance model in India shows that decentralisation can be effective if accompanied by institutional strengthening at the local level, as evidenced by successful state-level health interventions (21). In contrast, Germany's centralized model effectively manages institutional capacity at the national level, ensuring uniform policy implementation and a coordinated approach to health system strengthening (16).

This analysis builds on the work of Ciccone et al. (4), who highlight that governance mechanisms that align national and local health policies are essential for improving health system outcomes. Our findings suggest that institutional capacity must be bolstered across all levels of governance (national, provincial, and local) if health reforms are to be successfully implemented in LMICs.

### 6.2 Decentralisation: opportunities and challenges

The findings underscore the mixed results of decentralisation in LMICs, which corroborates the existing literature on the subject (24). While Germany benefits from a balanced decentralisation system that ensures local adaptability while maintaining central oversight, the decentralisation efforts in South Africa and India have yielded less favourable outcomes. South Africa's decentralisation, particularly in rural and underserved areas, has exacerbated regional disparities in health service delivery due to weak local governance and poor institutional coordination (22). Similarly, India's decentralisation has often resulted in fragmented health systems, where local governments lack the capacity to manage resources effectively, especially in less developed states (21).

These findings suggest that decentralisation can only be successful when accompanied by institutional strengthening at the local level. The successful examples of Rwanda's decentralised health services and Germany's hybrid decentralisation model highlight the importance of intergovernmental coordination and local capacity building (23). This also reinforces the argument made by Cobos Muñoz et al. (24) that decentralisation without adequate local capacity can worsen inequalities and undermine the performance of health systems.

Therefore, the findings challenge the prevailing assumption that decentralisation is inherently beneficial for improving health service delivery. Instead, they suggest that context-specific solutions, which balance central control with local autonomy, are more likely to improve health outcomes in LMICs.

### 6.3 Leadership and policy implementation

A central theme emerging from the findings is the crucial role of leadership in driving successful health reforms. The results confirm that strong leadership is consistently linked to effective policy formulation, intersectoral coordination, and sustained health system improvements (21, 22). Germany's leadership has been instrumental in ensuring policy coherence, while the leadership challenges in South Africa and India have resulted in policy fragmentation and implementation failures (22).

In South Africa, leadership fragmentation between national and provincial authorities has hindered the effective implementation of key reforms like the NHI (22). The lack of coordinated political commitment and accountability in India further exacerbates governance challenges, as leadership quality varies significantly across regions, with some states performing better than others in implementing health reforms (21).

These findings reinforce the centrality of leadership in the governance of health systems. As Gilson et al. (9) assert, leadership is not merely about individual competency but also about creating an environment where governance structures support policy coherence, resource allocation, and effective reform implementation. Therefore, investing in leadership capacity at all levels of government is essential for successful health system strengthening in LMICs.

### 6.4 Digital health integration: potentials and barriers

The adoption of digital health solutions is increasingly seen as a strategy for improving healthcare access and efficiency in LMICs. The results indicate that digital health integration has made substantial strides in Germany, where EHRs and telemedicine have enhanced care coordination and health service efficiency (26). In contrast, South Africa and India have faced significant barriers to digital health adoption, including infrastructure deficits, digital illiteracy, and inadequate funding (14, 28).

In India, mHealth solutions have shown promise in improving maternal health and disease prevention, but their impact is limited by regional disparities in infrastructure and digital literacy (14). Similarly, South Africa's digital health initiatives are constrained by infrastructural challenges in rural areas, limiting the effectiveness of these technologies in improving health outcomes across the population (25).

These findings contribute to the existing literature by emphasising that while digital health holds significant potential for improving healthcare delivery in LMICs, its success depends heavily on addressing infrastructure gaps and improving digital literacy (26). This insight extends the arguments of Wyber et al. (25) and Frost et al. (14), who highlight the need for context-sensitive strategies to integrate digital health into existing healthcare systems.

### 6.5 Limitations of the study

While the findings offer valuable insights, several limitations must be acknowledged. First, the study relies heavily on secondary data, which may introduce biases or gaps in the analysis. Although efforts were made to triangulate data sources and ensure the credibility of the findings, the reliance on existing literature limits the depth of primary data collection and real-time perspectives from policymakers and health professionals.

Second, interpretative biases may have influenced the analysis, particularly in the way governance models and health system reforms were framed. Despite efforts to maintain objectivity, the subjective nature of qualitative research means that different researchers may interpret the same data differently.

Finally, the study's comparative design has inherent limitations. Although the comparative case study approach allows for an in-depth understanding of health systems in diverse contexts, it is not always generalisable to other LMICs. The heterogeneity of contexts (in terms of political systems, cultural factors, and economic conditions) means that the findings may not be universally applicable.

This study provides a critical conceptualisation of the relationship between public administration and health system strengthening in LMICs. By integrating governance, leadership, institutional capacity, and digital health within a unified theoretical framework, it advances the understanding of the factors that influence health system performance in diverse LMIC contexts. Furthermore, the study's findings extend the literature by demonstrating the complex interdependencies between these elements and highlighting the context-specific nature of health system reforms.

## 7 Conclusion

This study has critically explored the role of public administration in health system strengthening (HSS) in LMICs through a comparative analysis of Germany, South Africa, and India. By applying Institutional Theory, the research has illuminated the centrality of governance structures, institutional capacity, leadership, decentralisation, and digital health integration in shaping the success or failure of health system reforms. The findings underscore that health system strengthening is not merely a technical or financial challenge, but an inherently institutional and administrative process. The application of Institutional Theory provides critical insights into how the institutional environment (comprising formal governance mechanisms, normative expectations, and informal administrative practices) shapes the effectiveness of health system reforms in LMICs. The study contributes to the growing body of literature that challenges the view that policy design alone determines health outcomes. Instead, it highlights that institutional coherence and governance quality are decisive in translating policy into tangible results. This theoretical lens shifts the focus from the technical and resource-based aspects of health system strengthening to a more comprehensive understanding that includes institutional alignment and administrative capacity. A key theoretical contribution of this study lies in the integration of governance and institutional capacity within a unified framework, offering a nuanced view of how public administration mediates health system performance. By critically engaging with these concepts, the study demonstrates that effective governance is not merely about structural design but is also deeply tied to institutional practices, leadership dynamics, and intersectoral coordination. This aligns with Gilson et al. (5), who emphasize that institutional structures must be context-sensitive and able to adapt to the local governance needs of health systems. Furthermore, the study highlights the critical need for contextual understanding when designing health reforms. As shown in the cases of South Africa and India, decentralisation and digital health adoption can fail when institutional capacity at the local level is insufficient. This challenges the prescriptive adoption of global health models without considering the unique political, social, and economic contexts of LMICs. By drawing on Institutional Theory, the study offers a theoretical framework that can guide comparative research on health systems, encouraging scholars to incorporate governance dynamics into their analyses.

### 7.1 Practical implications

From a practical standpoint, the findings of this study offer valuable policy insights for LMICs seeking to strengthen their health systems. The study's results reinforce the need for holistic reform strategies that consider both technical solutions (such as digital health technologies) and institutional factors (such as governance quality, leadership capacity, and accountability systems). In particular, the findings call for a more strategic approach to institutional capacity building, ensuring that health reforms are not only designed effectively but are also implemented within robust governance frameworks capable of fostering accountability and coordination.

For instance, the experience of Germany suggests that a centralised governance model can facilitate policy coherence, whereas the challenges faced by South Africa and India illustrate the importance of building local governance capacity before decentralising health services. Therefore, countries looking to implement decentralised models of governance should prioritise institutional strengthening at the local level, including investments in leadership development and resource management (4). Decentralisation should not be viewed as a one-size-fits-all solution, but as a complex process that requires careful consideration of local governance capacities and the specific needs of the population (24).

Additionally, the study highlights that digital health solutions, while promising, require significant infrastructure investments, training programs, and policy alignment to be effective in LMICs. Countries like South Africa and India can learn from Germany's successful integration of telemedicine and EHRs, adapting these technologies to their own resource-constrained contexts. However, this requires addressing the digital divide, particularly in rural areas, by focusing on digital literacy, internet accessibility, and inclusive health technology policies (14). The practical implications of these findings suggest that policymakers must take a systems thinking approach, recognising the interplay between governance, institutional capacity, and technological solutions in strengthening health systems.
